# Preparation and evaluation of conjugate nanogels of glycyl-prednisolone with natural anionic polysaccharides as anti-arthritic delivery systems

**DOI:** 10.1080/10717544.2020.1865478

**Published:** 2020-12-29

**Authors:** Kohei Mizuno, Yuri Ikeuchi-Takahashi, Yoshiyuki Hattori, Hiraku Onishi

**Affiliations:** Department of Drug Delivery Research, Hoshi University, Tokyo, Japan

**Keywords:** Conjugate nanogel, prednisolone, natural anionic polysaccharide, anti-arthritis, efficacy

## Abstract

Although prednisolone (PD) is used as an anti-arthritis drug due to its rapid and strong anti-inflammatory potential, its frequent and large dosing often brings about adverse effects. Therefore, targeting therapy has attracted increasing attention to overcome such adverse effects. In the present study, nanogels (NGs) composed of macromolecule–PD conjugates were developed as a novel targeting delivery system, and their anti-inflammatory potential was examined. Conjugates were prepared by carbodiimide coupling between glycyl-prednisolone (GP) and the natural anionic polysaccharides, alginic acid (AL) and hyaluronic acid (HA). NGs were produced by the evaporation of organic solvent from the conjugate solution. The obtained NGs, named AL-GP-NG and HA-GP-NG, respectively, were examined for particle characteristics, *in vitro* release, pharmacokinetics, and *in vivo* efficacy. Both NGs were several hundred nanometers in size, had negative zeta potentials, and several % (w/w) drug contents. They released PD gradually at pH 7.4 and 6. They exhibited fairly good retention in the systemic circulation. In the efficacy examination using rats with adjuvant-induced arthritis, both NGs showed the stronger and more prolonged suppression of paw inflammation than PD alone. These suggested that the present NGs should be possibly useful as anti-arthritis targeting therapeutic systems.

## 1. Introduction

Rheumatoid arthritis (RA) is a chronic autoimmune disease (Calabresi et al., 2018). It affects 0.5–1% of the population worldwide, particularly developed countries (Bax et al., 2011; Cassone et al., 2020). RA is characterized by joint inflammation and the progressive destruction of cartilage, and affects diarthrodial joints (Choy, 2012). This immune disorder primarily develops at synovial tissue, leading to inflammation and invasion of the synovium as well as the destruction of cartilage and bone (Andreas et al., 2008). Regarding its etiology, genetic and environmental factors are essentially associated with the induction of RA (Cojocaru & Chicoş, 2013; Glant et al., 2014).

In the treatment of RA, pharmacotherapy is very important. Commonly used medications include disease-modifying anti-rheumatic drugs, nonsteroidal anti-inflammatory drugs, glucocorticoids, and biologics (Boers et al., 1997; Ash & Emery, 2012; Yang et al., 2017; Abbasi et al., 2019). Although biologics such as antibody drugs, which are very effective, are becoming more widely used, they are associated with a risk of adverse effects and are expensive (Silva et al., 2010; Carter et al., 2012). Therefore, conventional drugs are still important and widely used in the treatment of RA.

Glucocorticoids are highly effective against RA (Walz et al., 1971). They suppress inflammation more rapidly and strongly than other types of drugs (Ward & Cloud, 1966). Prednisolone (PD), an anti-inflammatory steroidal agent, is widely used in the treatment of RA (Buttgereit et al., 2008, 2013). As PD exhibits hormonal activities in various tissues in the body; adverse effects have to be managed in its use. Frequent dosing with moderate and/or high amounts of PD often causes adverse effects, such as diabetes, osteoporosis, glaucoma, and arteriosclerosis (Yano et al., 2002; Wang et al., 2007; Onishi et al., 2008; Kong et al., 2009; Van den Hoven et al., 2011). However, when the dose amount and/or dosing frequency are reduced, its efficacy decreases. Therefore, the specific delivery of PD to the diseased site is required in order to resolve these issues.

In the present study, nanogels (NGs), composed of macromolecular prodrugs of PD, were developed. In general, a NG is a kind of nanoparticle. NGs are three-dimensional nano-sized hydrogels formed by physical or chemical crosslinking of polymers (Boridy et al., 2009; Bae & Na, 2010; Kim et al., 2019). NGs are not dissolved in water, but hydrophilic parts swell. Thus, NGs have both characteristics of hydrogels and nanoparticles. In the conjugates composed of water-soluble polymer and hydrophobic small molecules, their self-assembly is brought about among the hydrophobic ligands, leading to the formation of NGs in water. As to the detailed preparation of NGs, the solvent diffusion (Bae & Na, 2010; Onishi et al., 2019) and sonication method (Park et al., 2004; Boridy et al., 2009) have been reported. We previously reported the conjugate of chondroitin sulfate (CS) and glycyl-prednisolone (GP), named CS–GP, which formed the NG at the condition of PD content of more than 4% (w/w).

The targeting delivery toward RA is based on the following concept. Neovascular vessels, being highly permeable, grow well in inflammatory regions, allowing macromolecules and nanoparticles to leak into the diseased sites (Metselaar et al., 2003; Taylor & Sivakumar, 2005; Pandya et al., 2006). NGs, having nanoparticle characteristics, can be targeted to the inflammatory regions, which is similar to enhanced permeability and retention (EPR) effect in the tumor tissues (Matsumura & Maeda, 1986).

In the present study, we focused on alginic acid (AL) and hyaluronic acid (HA), which are actively examined in medical fields because of their highly biocompatible properties. AL is used as a biomaterial for wound dressings, etc. (Cabrales et al., 2005; Moscovici, 2015; Severino et al., 2019). HA is widely distributed in the connective tissues of the animal body, and is used as a therapeutic agent or material for drug carriers (Fraser et al., 1981; Hirakura et al., 2010; Kim et al., 2019). As these polymers are highly water-soluble, their conjugates with PD were considered to form the NGs due to the hydrophobicity of PD. As to previously reported CS–GP, the water-soluble type of CS–GP, injected intravenously, was eliminated fairly rapidly from the blood circulation, resulting in the fact that it did not exhibit so prolonged anti-inflammatory effects (Onishi et al., 2013, 2014); though the NG type of CS–GP was not tested. CS itself was reported to undergo a fairly rapid systemic clearance and degradation. Considering these features of CS and CS–GP, we here attempted to examine the potential of AL and HA as NG carriers for the targeting therapy against arthritis. Namely, the preparation of the conjugates of GP with AL and HA, and formation of their NGs, their *in vitro* properties and *in vivo* therapeutic profiles were demonstrated.

## 2. Materials and methods

### 2.1. Materials

Prednisolone, carbonyl-diimidazole (CDI), dimethyl-aminopyridine (DMAP), *N*-hydroxysuccinimide (NHS), 1-(3-dimethylaminopropyl)-3-ethylcarbodiimide hydrochloride (WSC), and sodium alginate (AL-Na; low viscosity grade (viscosity of 1% aqueous solution at 25 °C = 32.7 ± 0.4 mPa·s (*n* = 3)), cat no. 154725, lot no. QR13046) were purchased from FUJIFILM Wako Pure Chemical Corp. (Osaka, Japan). *N*-Trityl-glycine (Tr-G) was obtained from Sigma-Aldrich Co., LLC. (St. Louis, MO). Hyaluronic acid (viscosity of 1% aqueous solution at 25 °C = 1.2 ± 0.1 mPa·s (*n* = 3), product code H0595, lot no. 8FUKM-CI) was purchased from Tokyo Chemical Industry Co., Ltd. (Tokyo, Japan). Non-viable and desiccated *Mycobacterium tuberculosis* H37 Ra was obtained from Becton, Dickinson and Company (Franklin Lakes, NJ) and used in the preparation of adjuvants. All other chemicals were of reagent grade.

### 2.2. Animals

Female Lewis rats (160–170 g) were purchased from Charles River Laboratories Japan, Inc. (Yokohama, Japan). They were bred on the breeding diet MF (Oriental Yeast, Co., Ltd., Tokyo, Japan) with water *ad libitum*. Regarding the conditions of the animal room, temperature was set at 23 ± 1 °C, relative humidity was maintained at 60 ± 1%, and the light-dark cycle was 12 h. The experimental protocol was approved by the Committee on Animal Research of Hoshi University (Tokyo, Japan), in which the approval code was 30-076 (approval date: April 20 2018). Animal experiments were conducted based on the Guiding Principles for the Care and Use of Laboratory Animals of Hoshi University (Tokyo, Japan).

### 2.3. Preparation of conjugate nanogels

**Figure 1.**
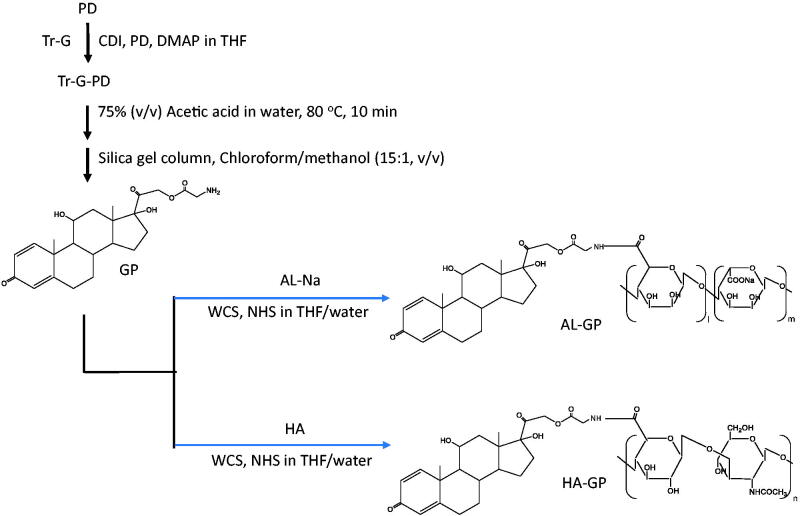
Synthesis of AL-GP and HA-GP.

The conjugates of GP with AL and HA, named AL-GP and HA-GP, respectively, were synthesized as shown in Figure 1. The detailed procedure was as follows. First, the synthesis of Tr-G ester of PD (Tr-GP) was conducted according to the method described by Onishi & Matsuyama (2013). First, Tr-G (477 mg, 1.5 mmol) and CDI (243 mg, 1.5 mmol) were stirred in 10 mL tetrahydrofuran (THF) at 0 °C for 30 min, and DMAP (15 mg, 0.12 mmol) and PD (270 mg, 0.75 mmol) were then added and stirred at room temperature for 4.5 h. After evaporation of the solvent, Tr-GP was recrystallized using methanol. The chemical structure of Tr-GP was confirmed by the measurements of proton nuclear magnetic resonance (^1^H NMR) and electron ionization mass spectrometry (EI-MS). ^1^H NMR (DMSO-d6), *δ* (ppm): 7.20–7.42 (m, 16H, Tr-G Tr-H, PD C1-H), 6.15–6.18 (d, 1H, PD C2-H), 5.92 (s, 1H, PD C4-H), 5.02–5.05 (d, 1H, PD C21-H), 4.72–4.75 (d, 1H, PD C21-H′), 4.29 (s, 1H, PD C11-H), 2.97–2.99 (m, 2H, Tr-G C-H2). EI-MS *m/z*: 659 (M+).

Next, GP was obtained by detritylation of Tr-GP: soon after Tr-GP (300 mg) was dissolved in 75% (v/v) aqueous acetic acid (10 mL) by heating at 80 °C for 10 min, triphenylcarbinol was precipitated. Then, the mixture underwent ice cooling and filtrated. The filtrate was evaporated with a rotary evaporator, and the residue was dissolved in the chloroform/methanol mixture. The resultant solution was chromatographed with a silica gel column using a chloroform/methanol mixture (15:1, v/v) as the elution solvent. The fractions of the major product (GP) were collected and evaporated to dryness. The structure of GP was confirmed by the measurements of ^1^H NMR and matrix-assisted laser desorption/ionization-time of flight mass spectrometry (MALDI-TOF-MS). ^1^H NMR (DMSO-d6), *δ* (ppm): 7.31–7.33 (d, 1H, PD C1-H), 6.15–6.17 (d, 1H, PD C2-H), 5.92 (s, 1H, PD C4-H), 4.97–5.00 (d, 1H, PD C21-H), 4.84–4.87 (d, 1H, PD C21-H′), 4.27–4.38 (m, 3H, glycine C-H2, PD C11-H). MALDI-TOF-MS *m/z*: 418.54 ([M + H]^+^).

Finally, the conjugates of GP with the polysaccharides were prepared as follows. As to AL-GP, after GP (30 mg) and AL-Na (60 mg) were dissolved in 10 mL of the THF/water (1:1, v/v) mixture, WSC (500 mg), and NHS (300 mg) were added, and the mixture was stirred at room temperature overnight. The resultant mixture was chromatographed with a Sephadex G50 gel column (2.6 cm (inner diameter)×19 cm (length)) using a mixture of 0.1 M NaCl aqueous solution and methanol (7:3, v/v) as the elution solvent. After the macromolecular fractions had been gathered, the solution obtained was dialyzed against water at 4 °C. The medium remaining in the dialysis bag was lyophilized to yield AL-GP powder. HA-GP was prepared in the same manner, except for the use of HA (60 mg) instead of AL-Na (60 mg).

**Figure 2.**
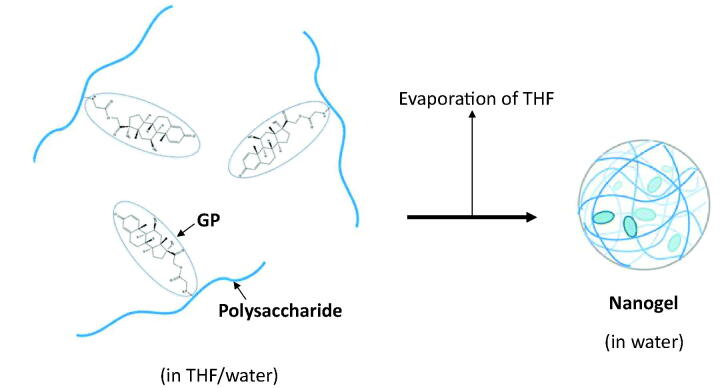
Schematic diagram of nanogel formation by organic solvent evaporation.

The NGs of AL-GP and HA-GP, named AL-GP-NG and HA-GP-NG, respectively, were obtained by the solvent evaporation technique (Figure 2). After the conjugate was dissolved in the mixture of THF and water, THF was evaporated gradually using a rotary evaporator *in vacuo* until THF was removed completely from the solvent. The NG was obtained as a white suspension in water.

### 2.4. Characterization of NGs

The drug contents of AL-GP-NG and HA-GP-NG were examined by the complete liberation of PD from NGs followed by spectrophotometric measurements. The NGs, AL-Na, and HA (3 mg) were placed in 5 mL of 0.1 M NaOH aqueous solution and incubated at 45 °C for 10 min. After the media were centrifuged, supernatants were measured spectrophotometrically at 246 nm. The difference in absorbance at 246 nm between that supernatant and polysaccharide solution was obtained as a net absorbance by the conjugated PD. The amount of the conjugated PD was calculated by comparing the net absorbance (246 nm) with the absorbance of PD itself in 0.1 M NaOH aqueous solution (246 nm).

The particle size and zeta potential of each NG were analyzed using the dynamic light scattering apparatus, ELS-Z2 (Otsuka Electronics Co., Ltd., Osaka, Japan).

### 2.5. *In vitro* drug release from NGs

AL-GP-NG and HA-GP-NG were suspended in phosphate-buffered saline of pH 7.4 (PBS) and 0.1 M acetate buffer of pH 6.0 at a concentration of 2 mg/mL. The suspension (4 mL) was placed in a cellulose dialysis tube (MW cutoff 14,000) made by Viskase Companies, Inc. (Darien, IL). The tube containing the NG sample was completely immersed in 16 mL of PBS or acetate buffer, and incubated at 37 °C by shaking horizontally at 60 rpm for 48 h. Aliquot samples (50 µL) were taken at appropriate time points. Immediately after sampling, 100 µL of 0.1 M acetate buffer (pH 4) was added to the sample to suppress further release, because the carboxy ester is very stable around pH 4. The resultant mixture was analyzed by HPLC to measure the concentration of released PD.

### 2.6. Plasma concentration studies after intravenous (i.v.) injection of NGs

AL-GP-NG and HA-GP-NG were suspended in saline at 2.5 mg PD eq./mL. The NG suspension was injected intravenously via the jugular vein into normal rats at 2.5 mg PD eq./kg. Blood samples (each 300 µL) were taken immediately before and 0.5, 1, 3, and 7 h after administration. After each sampling, centrifugation was performed at 1500 rpm to obtain plasma. Plasma (50 µL), 20% trichloroacetic acid (10 µL), and methanol (35 µL) were mixed and shaken vigorously for 5 min. After centrifugation of the mixture, the supernatant was analyzed by HPLC to assess the concentration of free PD.

The concentration of total (free + conjugated) PD was analyzed as follows: plasma (50 µL) and 0.1 M NaOH aqueous solution (50 µL) were mixed, and the mixture was incubated at 45 °C for 10 min. Then, 0.1 M HCl aqueous solution (50 µL) was added to neutralize the sample pH. Furthermore, 20% trichloroacetic acid (30 µL) and methanol (105 µL) added, and the mixture underwent vigorous shaking for 5 min. After centrifugation of the sample, the supernatant was analyzed by HPLC to determine the concentration of PD.

As the control, PD alone, dissolved in 50% (w/v) polyethylene glycol 400 (PEG400) saline solution at 2.5 mg/mL, was administered intravenously to normal rats via the jugular vein at 2.5 mg/kg. Blood sampling and analyses of the plasma concentration of PD were performed as in the case of the free PD in the NG suspension.

Pharmacokinetic parameters were calculated using the program MULTI produced by Yamaoka et al. (1981).

### 2.7. *In vivo* examination of efficacy and adverse effects in rats with adjuvant-induced arthritis

**Figure 3.**
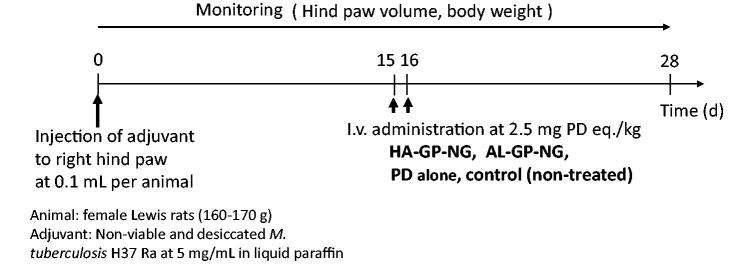
*In vivo* experimental schedules for examination of efficacy of conjugate nanogels.

*In vivo* experiments of efficacy and adverse effects were performed using rats with adjuvant-induced arthritis. The *in vivo* experimental schedules are shown in Figure 3. The arthritic animal models were produced according to the method described by Hirano et al. (1994) and Matsukuma et al. (2005). Non-viable and desiccated *M. tuberculosis* H37 Ra (20 mg) was suspended in 4 mL of liquid paraffin and ground in a mortar. The resultant suspension (100 µL) was injected intracutaneously into the foot pad of the right hind paw as the adjuvant. Body weights and both hind paw volumes were measured every day after the injection of the adjuvant.

Drugs (NGs or PD alone) were administered intravenously at 2.5 mg PD eq./kg 15 and 16 d after the adjuvant injection; the drug administration was conducted in the manner of consecutive dosing at 2.5 mg PD eq./kg × 2 d. The control group received no treatment. Body weights and the volumes of both hind paws were monitored every day until 28 d after the adjuvant injection. Changes in body weight were examined as an index of adverse effects. The hind paw volume was investigated as an index of the degree of inflammation.

### 2.8. HPLC assay

A HPLC analysis was performed using an apparatus made by Shimadzu Corp. (Kyoto, Japan); A LC-20AD pump, SPD-20A UV/VIS detector, SIL-20AC autosampler, SLC-10A system controller, and LabSolutions system were used at room temperature. An YMC-Pack ODS-AM column (6 mm (inner diameter)×150 mm (length)) was used as an analytical column. The detector was set at 246 nm.

In *in vitro* studies, a 30% (v/v) 2-propanol aqueous solution containing trifluoroacetic acid at 0.1% (w/v) was used as the mobile phase, and flowed at 1 mL/min. The sample (20 µL) was injected onto the column.

As to the analysis of samples in *in vivo* experiments, a mixture of acetonitrile and 50 mM sodium citrate–phosphoric acid buffer of pH 4.1 (35:65, v/v) was used as the mobile phase, and its flow rate was set at 1 mL/min. Each sample (20 µL) was injected on the column. The concentration of PD was calculated by the absolute calibration method.

### 2.9. Statistical analysis

Statistical analyses were performed with a one-way ANOVA followed by Tukey’s post hoc test, and the significance of differences was set as *p*< .05.

## 3. Results and discussion

### 3.1. Preparation and characteristics of NGs

Carboxy esterification between Tr-G and PD was conducted as described previously (Onishi & Matsuyama, 2013), with CDI in dry THF using DMAP as a catalyst, in which esterification at the position of C21 of PD was confirmed from the chemical shift at the C21 protons: (4.04–4.09, 4.47–4.51 ppm) in PD, (4.72–4.75, 5.02–5.05) in Tr-GP. Furthermore, the integrated intensities of the protons of Tr-GP showed that the ratio of glycine to PD was 1:1 (mol/mol). These indicated the ester formation at C21 of PD and the composition ratio of PD/Tr-G at 1:1 (mol/mol). GP, obtained by detritylation of Tr-GP, showed similar features for the chemical shift and proton integrated intensities. GP was confirmed to be a glycine monoester of PD, in which the ester was formed at the C21 position.

Conjugates of GP with the natural anionic polysaccharides, AL and HA, called AL-GP and HA-GP, respectively, were prepared by carbodiimide coupling (Figure 1). Nanogels composed of conjugates, named AL-GP-NG and HA-GP-NG, respectively, were readily prepared by the evaporation of organic solvent from the conjugate solution in the THF/water mixture (Figure 2). In the preliminary studies, the white suspension by the NG formation was recognized at the PD content of 3% (w/w) or more, while such a white suspension state was not observed at the PD content of less than 1% (w/w).

**Figure 4.**
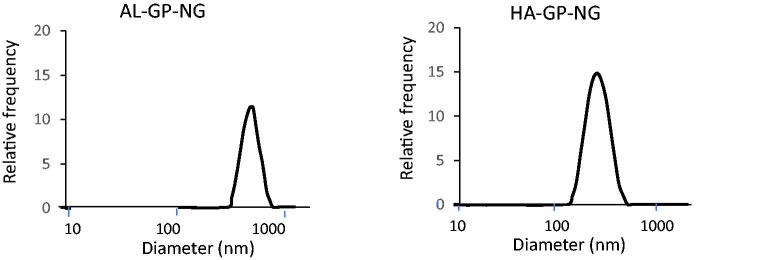
Size distributions of conjugate nanogels.

The PD content of the NG was determined from the absorbance at 246 nm after the alkaline hydrolysis of the ester bond. Although the treatment of PD in the alkaline solution gave some degradation of PD, the absorbance of the treated sample at 246 nm was hardly changed. Therefore, even if the PD was degraded to a fair extent in the complete liberation of PD from NG at the alkaline conditions, the drug content could be determined from the absorbance at 246 nm. The PD contents, particle sizes, and zeta potentials were obtained as shown in Table 1. Both NGs had several % (w/w) PD contents and submicron particle sizes. Figure 4 shows the particle size distribution of NGs, indicating that each NG had a simple size distribution. HA-GP-NG was slightly smaller than AL-GP-NG. Particle sizes may depend on the polysaccharide structure. Since AL has block structures, its sugar chain is not as flexible, which may inhibit compaction by hydrophobic interactions among PD moieties. On the other hand, since the sugar chain of HA is highly flexible, it allows for maximum hydrophobic interactions among PD moieties, which may lead to the better compaction of HA-GP-NG. Each NG had an anionic charge; however, the negative charge was not strong. These zeta potentials were considered to be adequate for blood compatibility and systemic retention (Ren et al., 2019).

**Table 1.** Preparation of conjugate nanogels and their particle features.

Nanogel	AL-Na (mg)	HA (mg)	GP (mg)	WSC (mg)	NHS (mg)	PD content (%, w/w)	Particle size^a^ (nm)	Zeta potential^a^ (mV)
AL-GP-NG	60	–	30	500	300	3.4	444 ± 88	–24.9 ± 0.6
HA-GP-NG	–	60	30	500	300	5.6	304 ± 63	–19.3 ± 0.6

^a^ The results are expressed as the mean ± S.D. (*n* = 3).

### 3.2. PD release from NGs

**Figure 5.**
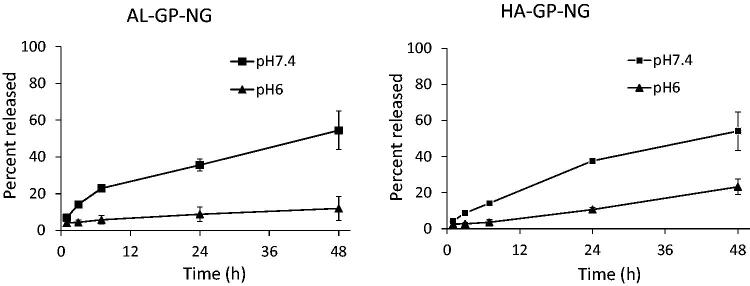
*In vitro* release of PD from conjugate nanogels at pH 6 and 7.4 at 37 °C. The results are expressed as the mean ± S.D. (*n* = 3).

The release profiles of PD from both NGs were obtained as shown in Figure 5. PD was gradually released at pH 6 and 7.4. In both NGs, the release rate was accelerated by an increase in pH, which was because the carboxy ester should be hydrolyzed faster at the basic pH. In both NGs, PD was released gradually at pH 7.4, at which approximately 60% was released at 48 h. At the pH 6, the release of PD was suppressed. Therefore, each NG considered to release PD gradually in the systemic conditions, resulting in the accumulation of NG into the inflammatory sites through the high permeability of the neovascular vessels. Furthermore, PD was released more slowly in the inflammatory sites due to their lower pH, which was reported to be 6–7.4 (Goldie & Nachemson, 1969). Accordingly, PD was considered to be localized to the inflammatory regions and to function for a prolonged period.

### 3.3. Plasma concentration–time profiles after i.v. injections of NGs

In order to find out the pharmacokinetic features of AL-GP-NG and HA-GP-NG, they were intravenously injected into normal rats, and the plasma concentration–time profiles of free PD and total (conjugated + free) PD were investigated.

Total PD was assessed from PD liberated from NGs by hydrolysis with 0.1 M NaOH. However, liberated PD was not fully recovered by HPLC because PD was decomposed at the strong basic pH. When AL-GP-NG and HA-GP-NG were treated in 0.1 M NaOH aqueous solution at 45 °C for 10 min, the mean recovery ratios of intact PD were 55 and 70%, respectively. The recovery ratios were used as correction coefficients to calculate total PD concentrations.

**Figure 6.**
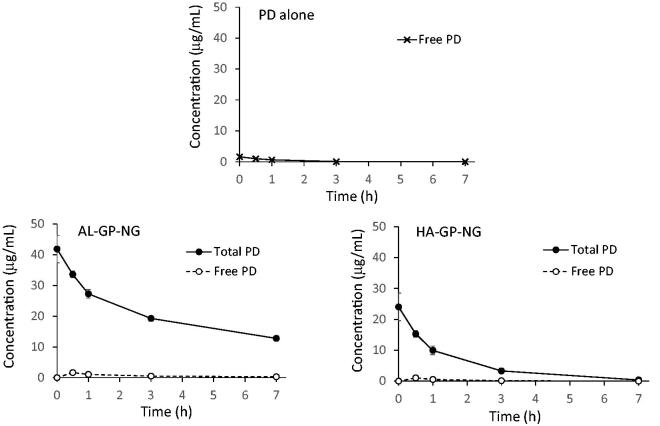
Plasma concentration–time profiles of free and total (free + conjugated) PD after i.v. administration of conjugate nanoparticles in normal rat. The results are expressed as the mean ± S.E. (*n* = 3).

Plasma concentration–time profiles were obtained as shown in Figure 6. Concentrations at time 0 h were calculated from extrapolations by mono-exponential curve fitting using the values at 0.5 and 1 h. Regarding the i.v. injection of PD alone, the plasma levels detected were very small. The mean plasma level was calculated to be less than 2 µg/mL immediately after the i.v. administration.

Regarding the i.v. injection of AL-GP-NG, the total PD concentration was markedly higher than the free PD concentration. The total PD concentration was higher than 40 µg/mL immediately after its administration, and was still higher than 10 µg/mL at 7 h. The free PD concentration was maximal at 0.5 h, being 1.64 µg/mL, and then the free PD concentration gradually decreased.

As to the i.v. injection of HA-GP-NG, total PD concentrations were also higher than free PD concentrations. The total PD level at 0 h was calculated at 24.03 µg/mL. Overall, total PD levels were lower with HA-GP-NG than with AL-GP-NG. At 7 h, the total PD concentration decreased to 0.39 µg/mL. The concentration of free PD was maximal at 0.5 h, being 1.05 µg/mL, and was not detected at 7 h. The levels of free PD were also lower with HA-GP-NG than with AL-GP-NG.

The pharmacokinetic parameters of AL-GP-NG and HA-GP-NG are shown in Tables 2 and 3, respectively. Free PD by PD alone, free PD by NG, and total PD by NG were compared. In AL-GP-NG, total PD gave markedly higher *C*_max_, AUC (0–7 h), MRT (0–7 h), and VRT (0–7 h) than free PD by PD alone and NG. Higher MRT (0–7 h) and VRT (0–7 h) were observed in free PD with AL-GP-NG than in free PD by PD alone. HA-GP-NG also showed similar pharmacokinetic features to those of AL-GP-NG. Since both NGs gave higher total plasma concentrations and more prolonged systemic retention, suggesting that the NGs should work well as a targeting delivery system to the sites of inflammation.

**Table 2.** Pharmacokinetic parameters of free and total PD in i.v. administration of AL-GP-NG to rats.

Preparation	Species	*C*_max_ (µg/mL)	*T*_max_ (h)	AUC (0–7 h) (µg·h/mL)	MRT(0–7 h) (h)	VRT (0–7 h) (h^2^)
PD alone	Free PD	1.61 ± 0.30	0.0 ± 0.0	1.91 ± 0.08	0.87 ± 0.05	0.67 ± 0.08
AL-GP-NG	Free PD	1.64 ± 0.03	0.5 ± 0.0	4.16 ± 0.01	2.41 ± 0.03**	4.22 ± 0.18**
	Total PD	41.84 ± 4.45**^,##^	0.0 ± 0.0	144.69 ± 4.97**^,##^	2.72 ± 0.04**^,##^	5.06 ± 0.06**^,##^

The results are expressed as the mean ± S.E. (*n* = 3).

** *p*< .01 vs. free PD (PD alone).

## *p*< .01 vs. free PD (AL-GP-NG).

**Table 3.** Pharmacokinetic parameters of free and total PD in i.v. administration of HA-GP-NG to rats.

Preparation	Species	*C*_max_ (µg/mL)	*T*_max_ (h)	AUC (0–7 h) (µg·h/mL)	MRT (0–7 h) (h)	VRT (0–7 h) (h^2^)
PD alone	Free PD	1.61 ± 0.30	0.0 ± 0.0	1.91 ± 0.08	0.87 ± 0.05	0.67 ± 0.08
HA-GP-NG	Free PD	1.05 ± 0.10	0.5 ± 0.0	1.61 ± 0.18	1.37 ± 0.16*	0.97 ± 0.18
	Total PD	24.03 ± 3.08**^,##^	0.0 ± 0.0	36.77 ± 3.91**^,##^	1.38 ± 0.08*	1.86 ± 0.08**^,##^

The results are expressed as the mean ± S.E. (*n* = 3).

* *p*< .05 vs. free PD (PD alone).

** *p*< .01 vs. free PD (PD alone).

## *p*< .01 vs. free PD (HA-GP-NG).

### 3.4. Efficacy of NGs in rats with adjuvant-induced arthritis

Efficacy studies were performed using diseased model rats with adjuvant-induced arthritis. Changes in body weight after i.v. administration were examined as adverse effects. The anti-inflammatory effectiveness of each drug was evaluated from changes in hind paw volumes (Metselaar et al., 2003; Quan et al., 2010; Aono & Sasano, 2018). The body weight ratio and hind paw volume ratio were calculated using the following equations:

(1) Body weight ratio on day X=(body weight on day X)/(body weight on day 15)

(2) Hind paw volume ratio on day X=(hind paw volume on day X)/(hind paw volume on day 15)

**Figure 7.**
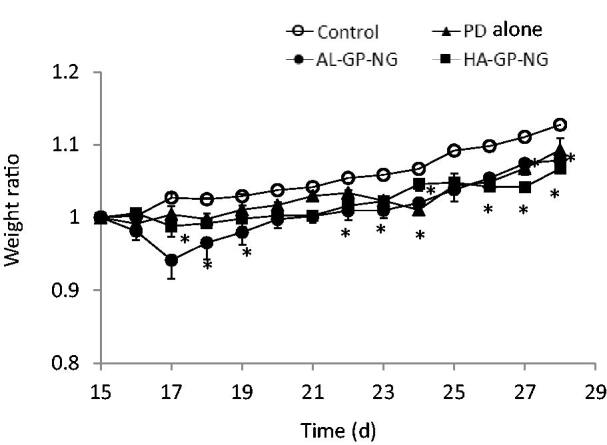
Change in body weight after i.v. administration of conjugate nanoparticles in rats with adjuvant-induced arthritis. The results are expressed as the mean ± S.E. (*n* = 3). *p< .05 vs. control.

**Figure 8.**
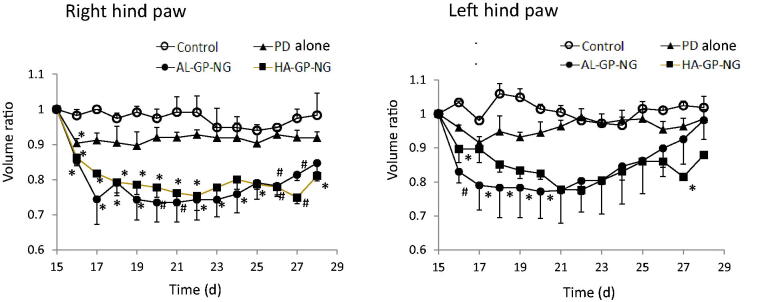
Change in body weight after i.v. administration of conjugate nanoparticles in rats with adjuvant-induced arthritis. The results are expressed as the mean ± S.E. (*n* = 3). *p< .05 vs. control and ^#^*p*< .05 vs. PD alone.

The body weight change, obtained by Equation (1), is shown in Figure 7. Although PD alone and NGs induced a small decrease in body weight in the early periods, this reduction quickly recovered. The anti-inflammatory effects were evaluated from the hind paw volume ratios (Equation (2)). The results obtained are shown in Figure 8. Although PD alone only slightly decreased the paw volume, its anti-inflammatory effects were not strong and did not significantly differ from those of the control group. On the other hand, both NGs strongly suppressed the volumes of both hind paws. In both NGs, stronger suppressive effects were observed in the right hind paw for long periods. Also, both NGs suppressed the left hind paw volume better than PD alone. The suppressive effects were not so different between AL-GP-NG and HA-GP-NG. As far as the profiles of the hind paw volume ratios were concerned, the present NGs appeared to show longer suppression of inflammation than the previously reported CS–SP conjugate (Onishi et al., 2013). The high efficacy of NGs against arthritis was considered to be due to their targeting ability to diseased sites. Targeting potential to arthritic sites will be elucidated in future studies.

## 4. Conclusions

In the present study, the natural anionic polysaccharides, AL and HA, were derivatized by carbodiimide coupling with GP. The conjugates obtained formed NGs in water and were named AL-GP-NG and HA-GP-NG, respectively. Both NGs were several hundred nanometers in size, had negative zeta potentials, and several % (w/w) drug contents. They gradually released PD at approximately 30-40% per day at pH 7.4 and more slowly at pH 6. Furthermore, both NGs exhibited fairly good retention in the systemic circulation. These *in vitro* and *in vivo* features suggested that both NGs should have good targeting potential to arthritic sites through the high permeability of the neovascular vessels of the inflammatory sites. In the efficacy studies using rats with adjuvant-induced arthritis, both the present NGs showed the strong and prolonged suppression of hind paw inflammation. Therefore, it was demonstrated that these NGs should be possibly useful in the treatment of arthritis.
